# Out-of-Hospital Cardiac Arrest Before and During the COVID-19 Pandemic in Hong Kong: Registry-Based Study From 2017 to 2023

**DOI:** 10.2196/56054

**Published:** 2024-05-21

**Authors:** Richard Huan Xu, Ruiqi Sun, Siu-Ngor Fu

**Affiliations:** 1 Department of Rehabilitation Sciences Faculty of Health and Social Sciences Hong Kong Polytechnic University Kowloon China (Hong Kong)

**Keywords:** out-of-hospital cardiac arrest, OHCA, COVID-19, pandemic, survival, Chinese, Asian

## Abstract

**Background:**

The COVID-19 pandemic has exerted a significant toll on individual health and the efficacy of health care systems. However, the influence of COVID-19 on the frequency and outcomes of out-of-hospital cardiac arrest (OHCA) within the Chinese population, both before and throughout the entire pandemic period, remains to be clarified.

**Objective:**

This study aimed to fill the gaps by investigating the prevalence and outcomes of OHCA in Hong Kong (HK) both before and during the whole pandemic period.

**Methods:**

This is a retrospective regional registry study. The researchers matched OHCA data with COVID-19–confirmed case records between December 2017 and May 2023. The data included information on response times, location of OHCA, witness presence, initial rhythm, bystander cardiopulmonary resuscitation (CPR), use of public-access defibrillation, resuscitation in the accident and emergency department, and survival to admission. Descriptive analyses were conducted, and statistical tests such as analysis of variance and *χ*^2^ were used to examine differences between variables. The incidence of OHCA and survival rates were calculated, and logistic regression analysis was performed to assess associations. The prevalence of OHCA and COVID-19 during the peak of the pandemic was also described.

**Results:**

A total of 43,882 cases of OHCA were reported in HK and included in our analysis. Around 13,946 cases were recorded during the prepandemic period (2017-2019), and the remaining 29,936 cases were reported during the pandemic period (2020-2023). During the pandemic period, the proportion of female patients increased to 44.1% (13,215/29,936), and the average age increased slightly to 76.5 (SD 18.5) years. The majority of OHCAs (n=18,143, 61.1% cases) occurred at home. A witness was present in 45.9% (n=10,723) of the cases, and bystander CPR was initiated in 44.6% (n=13,318) of the cases. There was a significant increase in OHCA incidence, with a corresponding decrease in survival rates compared to the prepandemic period. The location of OHCA shifted, with a decrease in incidents in public places and a potential increase in incidents at home. We found that CPR (odds ratio 1.48, 95% CI 1.17-1.86) and public-access defibrillation (odds ratio 1.16, 95% CI 1.05-1.28) were significantly associated with a high survival to admission rate during the pandemic period. There was a correlation between the development of OHCA and the prevalence of COVID-19 in HK.

**Conclusions:**

The COVID-19 pandemic has had a significant impact on OHCA in HK, resulting in increased incidence and decreased survival rates. The findings highlight the importance of addressing the indirect effects of the pandemic, such as increased stress levels and strain on health care systems, on OHCA outcomes. Strategies should be developed to improve OHCA prevention, emergency response systems, and health care services during public health emergencies to mitigate the impact on population health.

## Introduction

Out-of-hospital cardiac arrest (OHCA) refers to the sudden cessation of cardiac activity outside a hospital setting. It is a critical medical emergency that poses significant challenges to individuals’ health and health care systems. The escalating prevalence of OHCA in recent years has become a pressing concern. OHCA can affect individuals across all age groups and backgrounds, making it a universal health issue. According to data from 2020, the incidence of OHCA ranges from 30.0 to 97.1 per 100,000 population, with survival to hospital discharge varying from 3.1% to 20.4% worldwide [1]. This highlights the urgent need for effective preventive measures and improved emergency response systems.

The COVID-19 pandemic was associated with an increased incidence of several diseases. The stress on health care systems, disruptions to health care services, and changes in lifestyle and behavior due to lockdown measures contributed to these effects. Some diseases that showed an increased incidence included mental health disorders, cardiovascular diseases, and respiratory diseases. The COVID-19 pandemic negatively affected all components of systems of care related to OHCA, disrupting the chain of survival. A recent review demonstrated an increase of approximately 120% in the incidence of OHCA and a 65% decrease in OHCA-related survival to hospital discharge during the pandemic, compared to the prepandemic period [2].

In addition, previous studies have reported significant changes in the incidence and epidemiological characteristics of OHCA and the prognosis of patients with OHCA during the pandemic compared to the pre–COVID-19 period. These reports have indicated a decrease in bystander cardiopulmonary resuscitation (CPR) and public-access defibrillator (PAD) use. In contrast, the emergency medical service (EMS) reaction time, frequency of arrests at home, and use of supraglottic airways have increased. Treatment-induced return of spontaneous circulation, survival to admission (STA), survival to discharge, 30-day survival, and favorable neurological outcomes all showed decreased incidence during the pandemic compared with the prepandemic period [2-5]. Despite the end of the COVID-19 pandemic, comprehensive analyses of the entire period are scarce. This lack of evaluation of the effects of COVID-19 on OHCA during each wave of the outbreak may skew the results.

While many studies have explored the effect of COVID-19 on OHCA during the pandemic, 2 research gaps need to be filled. First, a significant limitation is that no studies have comprehensively monitored changes in the prevalence of OHCA in the population during the prepandemic period and during the entire duration of the COVID-19 pandemic. The lack of continuous monitoring data hinders our ability to fully comprehend the impact of the progression of the COVID-19 pandemic on OHCA. Second, there is a notable deficiency in data concerning the epidemiology of OHCA and the corresponding survival rates within the Hong Kong (HK) population. HK, a highly developed region of China, exhibits a lower survival rate for patients with OHCA compared to other industrialized countries [1]. A previous study reported a local survival rate of 15.3% upon admission and a discharge rate of 2.3% for patients with OHCA [6]. The lack of comprehensive data on OHCA in HK during the pandemic hampers our ability to fully understand the scope of the problem and develop effective strategies to improve survival rates for patients with OHCA. It is crucial to conduct extensive research to gather detailed epidemiological data on OHCA in the HK population. This would provide valuable insights into the impact of COVID-19 on OHCA and inform the development of targeted interventions to improve patient outcomes [1,6]. Therefore, we aimed to conduct a large-scale regional registry study to systematically explore the incidence and outcomes of OHCA during the prepandemic and pandemic periods in HK.

## Methods

### Study Design and Participants

We established our database by matching OHCA data, based on electronic records provided by the Hong Kong Fire Service Department (HKFSD), with confirmed COVID-19 case records from open-source data [7] provided by the Hong Kong Centre for Health Protection (CHP). The HKFSD is the official sector that provides publicly funded paramedic emergency ambulance services to more than 7.4 million HK residents, operating from 41 ambulance depots. It maintains the electronic records of individuals with OHCA who call for ambulance services. These records include call triage and dispatch information, EMS response times, demographics, first aid provided, and short-term clinical outcomes. From January 2020, the CHP began to report the official number of confirmed cases of infection with SARS-CoV-2 variants in HK. Information on the number of cases and daily deaths due to COVID-19 in HK at the peak of the pandemic was obtained from the Hong Kong University information hub [8].

### Data and Procedures

The HKFSD provided general data on OHCA cases through its internal record system. The research team collaborated with a superintendent from the HKFSD. This superintendent, who is in charge of quality assurance and ambulance services training, was appointed by the chief director of the HKFSD to provide the necessary data. The data elicitation method followed the method used in a previous study aimed at exploring the prevalence of OHCA in HK [6]. The senior author of that paper (YC Siu) is a member of our research team. A list of variables of interest developed based on internal discussion and a literature review was provided to the superintendent for internal discussion. The research team, superintendent, and senior medical officers from the HKFSD held 3 rounds of meetings to revise and confirm the data analysis protocol and the variable list. This study included patients of all ages. Victims of OHCA who were directly transferred to the public mortuary from the scene by EMS personnel and patients who did not use a ground ambulance were excluded from this study.

We collected data from December 1, 2017, to May 31, 2023. These data included the date of the incident, the response time (from the time of the call to the arrival of an ambulance or EMS personnel), the location of the OHCA (home, en route to hospital, nursing home, public place, or street), and the presence of an OHCA witness (yes or no). Clinical data consisted of the initial rhythm (pulseless electrical activity, ventricular fibrillation or ventricular tachycardia, asystole, or other), bystander CPR (yes or no), the number of times a PAD was used, resuscitation in the accident and emergency (A&E) department (yes or no), and STA (yes or no). Sociodemographic data included sex, age, and area of residence.

Although cases of COVID-19 were first reported in late December 2019 in Wuhan, the HK CHP started recording such cases in HK in January 2020. Therefore, we defined the prepandemic period as December 1, 2017, to December 31, 2019, and the pandemic period as January 1, 2020, to May 31, 2023, despite the World Health Organization announcing the end of the pandemic on May 5, 2023. We compared patient characteristics, the setting of OHCA occurrences, and OHCA outcomes between the prepandemic and pandemic periods.

### Statistical Analysis

Descriptive analyses were primarily used to present our findings. Continuous data are presented as means with SDs, while categorical data are presented as frequencies (n) and proportions (%). The differences between continuous variables were examined using analysis of variance, the Mann-Whitney test, or the Kruskal-Wallis test, as appropriate. Differences between categorical variables were examined using the *χ*^2^ test.

The total incidence of OHCA (‰) in HK and the incidence stratified by 3 districts (Hong Kong Island, Kowloon, and the New Territories) are presented as the number of OHCA cases divided by the mid-year population and survival to A&E department admission (%) from 2018 to 2022, respectively. Figures were created to visually represent the changing incidence of OHCA across different years and its relationship with the outbreak and progression of the COVID-19 pandemic. Additionally, we present the change in the prevalence of OHCA at different key cutoff points (eg, different waves of an outbreak) of the COVID-19 pandemic in HK.

Multivariate logistic regression analysis was used to assess the association between survival and A&E department admission, as well as the use of CPR and PAD during the prepandemic and pandemic periods. The analysis was adjusted for factors including sex, age, the cause of OHCA, the initial rhythm, the response time, whether the OHCA was witnessed, the location of the OHCA, and the area of residence of the patient. Adjusted odds ratios (aORs) and their 95% CIs were calculated. Furthermore, we collected additional data and described the prevalence of OHCA and COVID-19 during the peak of the pandemic (11 weeks from February 7, 2022, to April 24, 2022). Differences with *P* values less than .05 were considered statistically significant. All statistical analyses were performed using Stata (version 16; StataCorp LLC).

### Ethical Considerations

The Institutional Review Board of Hong Kong Polytechnic University approved this study protocol (HSEARS 20211004004). The requirement for informed consent was waived.

## Results

### Participants’ Background Characteristics

From December 1, 2017, to May 31, 2023, HK reported 43,882 cases of OHCA (Table 1). Of these, 13,946 cases were recorded during the prepandemic period (2017-2019), and the remaining 29,936 cases were reported during the pandemic period (2020-2023). During the prepandemic period, 56.6% (7896/13,946) of the patients were men, with an average age of 75.81 (SD 17.12) years. The majority of OHCAs (n=7566 cases, 54.3%) occurred at home. During the pandemic period, the proportion of female patients increased to 44.1% (13,215/29,936), and the average age increased slightly to 76.5 (SD 18.5) years. The majority of OHCAs (n=18,143, 61.1% cases) occurred at home. A witness was present in 45.9% (n=10,723) of the cases, and CPR was initiated in 44.6% (n=13,318) of the cases. The average response time increased to 13.6 (SD 13.3) minutes, and only 8.3% (n=2464 cases) of the patients were alive when admitted to an A&E department, which is half the rate observed in the prepandemic period.

**Table 1:** Background characteristics of individuals and the outcome of OHCA^a^.

Characteristics		Prepandemic (December 2017 to December 2019)	Pandemic (January 2020 to May 2023)	*P* value	
**Sex, n/N (%)**					.13
	Male	7896/13,946 (56.6)	16,721/29,936 (55.9)		
	Female	6050/13,946 (43.4)	13,215/29,936 (44.1)		
**Region, n/N (%)**					.24
	Hong Kong Island	2679/13,946 (19.2)	5568/29,985 (18.6)		
	Kowloon	4992/13,946 (35.8)	10,747/29,985 (35.8)		
	New Territories and Outlying Islands	6275/13,946 (45)	13,670/29,985 (45.6)		
**Location of OHCA, n/N (%)**					<.001
	Street	622/13,944 (4.5)	1077/29,715 (3.6)		
	Home	7566/13,944 (54.3)	18,143/29,715 (61.1)		
	En route to a hospital	692/13,944 (5)	1366/29,715 (4.6)		
	Home for aged	3439/13,944 (24.7)	6475/29,715 (21.8)		
	Public places	1625/13,944 (11.7)	2654/29,715 (8.9)		
**Witnessed arrest, n/N (%)**					.001
	No	9165/13,946 (65.7)	19,179/29,902 (64.1)		
	Yes	4781/13,946 (34.3)	10,723/29,902 (35.9)		
**Initial electrocardiogram rhythm, n/N (%)**					<.001
	Asystole	11,098/13,942 (79.6)	24,327/29,875 (81.4)		
	Pulseless electrical activity	1880/13,942 (13.5)	3730/29,875 (12.5)		
	VF/VT^b^	937/13,942 (6.7)	1712/29,875 (5.7)		
	Others	27/13,942 (0.2)	106/29,875 (0.4)		
**Apparent cause of arrest, n/N (%)**					<.001
	Trauma	659/13,946 (4.7)	1128/29,972 (3.8)		
	Nontrauma	13,287/13,946 (95.3)	28,844/29,972 (96.2)		
**Resuscitation done in A&E^c^, n/N (%)**					<.001
	No	6780/13,946 (48.6)	4264/8068 (52.9)		
	Yes	7166/13,946 (51.4)	3804/8068 (47.1)		
**Bystander CPR^d^, n/N (%)**					<.001
	No	9308/13,945 (66.7)	16,534/29,852 (55.4)		
	Yes	4637/13,945 (33.3)	13,318/29,852 (44.6)		
**Bystander PAD^e^, n/N (%)**					.37
	No	13,665/13,946 (98)	29,423/29,985 (98.1)		
	Yes	281/13,946 (2)	562/29,985 (1.9)		
**STA^f^, n/N (%)**					<.001
	No	11,478/13,946 (82.3)	27,309/29,773 (91.7)		
	Yes	2468/13,946 (17.7)	2464/29,773 (8.3)		
Response time (min), mean (SD)		10.6 (7.8)	13.6 (13.3)	<.001	
Age (years), mean (SD)		75.8 (17.1)	76.5 (18.5)	<.001	

^a^OHCA: out-of-hospital cardiac arrest.

^b^VF/VT: ventricular fibrillation or ventricular tachycardia.

^c^A&E: accident and emergency department.

^d^CPR: cardiopulmonary resuscitation.

^e^PAD: public-access defibrillation.

^f^STA: survival to admission.

### Prevalence of OHCA During the COVID-19 Pandemic

**Figure 1:**
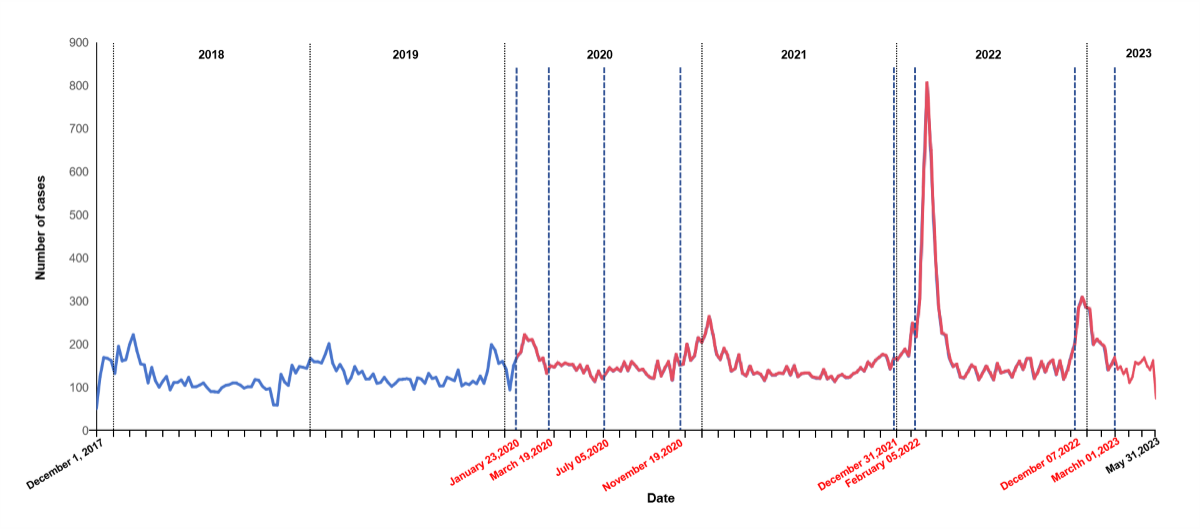
Prevalence of OHCA in HK from December 2017 to May 2023 with key local time points of COVID-19 pandemic. January 23, 2020: first wave of the COVID-19 outbreak. March 19, 2020: second wave of the COVID-19 outbreak. July 5, 2020: third wave of the COVID-19 outbreak. November 19, 2020: fourth wave of the COVID-19 outbreak. December 31, 2021: fifth wave of the COVID-19 outbreak. February 5, 2022: Chinese New Year Festival holiday. December 7, 2022: zero-tolerance measures are announced to be over in Mainland China. March 1, 2023: all mandatory mask-wearing requirements are lifted in Hong Kong. Blue line indicates prepandemic period. Red line indicates pandemic period. HK: Hong Kong; OHCA: out-of-hospital cardiac arrest.

Figure 1 presents a comparative analysis of the prevalence of OHCA during this study’s period. The prevalence of OHCA was lower in 2018-2019 than in 2020-2023. During the pandemic, each wave of the COVID-19 outbreak in HK aligned with a significant increase in the prevalence of OHCA. Notably, the peak in OHCA incidents coincided with the onset of the Chinese New Year holiday in 2022, a surge that lasted for approximately 11 weeks. This period marked the highest recorded number of COVID-19 cases throughout the pandemic. We also found a significant increase in the prevalence of OHCA following the discontinuation of zero-tolerance measures in mainland China. However, the removal of mandatory mask-wearing regulations by the HK government in March 2023 did not result in a significant increase in the prevalence of OHCA.

### OHCA Incidence Stratified by Year and Region

**Figure 2:**
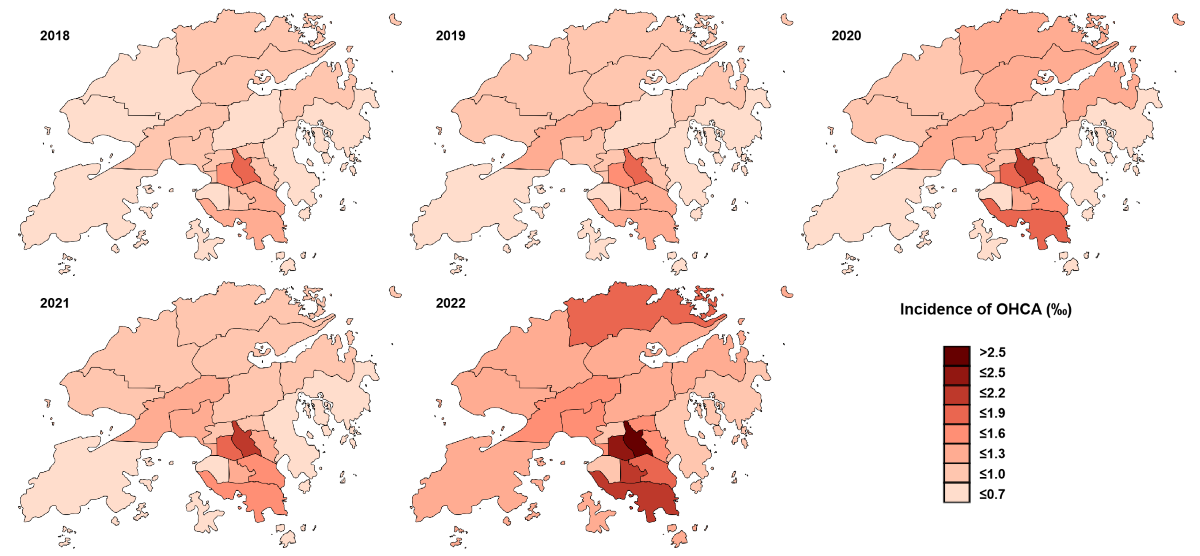
Surge of OHCA incidence across HK districts pre- (2018 and 2019) and during pandemic (2020-2022). HK: Hong Kong; OHCA: out-of-hospital cardiac arrest.

Table 2 demonstrates the increased incidence of OHCA from 2018 (6438/7,451,500, 0.85‰) to 2022 (10,618/7,345,000, 1.45‰). However, the STA rate significantly decreased from 16.9% (1090/6438) to 5.6% (584/10,424) during this period. When stratified by region, the incidence of OHCA was higher in Kowloon and Hong Kong Island than in the New Territories. During the prepandemic period, the STA rate was approximately 4% higher in the New Territories than in the other 2 districts. However, during the pandemic period, the gap in STA rates narrowed, with the STA rate in the New Territories becoming slightly lower than the STA rates in Kowloon in 2021 (245/3407, 7.2%, vs 212/2805, 7.6%) and 2022 (277/4837, 5.7%, vs 215/3636, 5.9%). Figure 2 provides additional information on the incidence of OHCA in all 18 districts. Generally, the incidence of OHCA was lower in districts in the north (New Territories) than in the other districts. Kwun Tong District (Kowloon, mid-part of HK) reported the highest incidence of OHCA during both the prepandemic and pandemic periods. The OCHA incidents, STA rate, and use of CPR among different demographic groups in the 3 regions over this study’s period are detailed in Multimedia Appendix 1.

**Table 2:** OHCA^a^ incidence, STA^b^, and CPR^c^ ratio in HK^d^ and stratified by regions.

Characteristics		Regions						
Year		KW^e^		HKI^f^		NT^g^		Overall
**OHCA incidence, n (‰)**								
	2018	2338 (1.03)	1232 (0.98)		2868 (0.73)		6438 (0.86)	
	2019	2398 (1.04)	1314 (1.05)		3119 (0.79)		6831 (0.91)	
	2020	2922 (1.27)	1504 (1.23)		3642 (0.92)		8068 (1.08)	
	2021	2818 (1.26)	1468 (1.23)		3410 (0.86)		7696 (1.04)	
	2022	3695 (1.68)	1978 (1.71)		4945 (1.24)		10,618 (1.45)	
**STA, n (%)**								
	2018	342 (14.6)	196 (15.9)		552 (19.2)		1090 (16.9)	
	2019	369 (15.4)	225 (17.1)		667 (21.4)		1261 (18.5)	
	2020	348 (11.9)	181 (12)		570 (15.7)		1099 (13.6)	
	2021	212 (7.6)	84 (5.7)		245 (7.2)		541 (7)	
	2022	215 (5.9)	92 (4.7)		277 (5.7)		584 (5.6)	
	2023	99 (7.5)	31 (5)		110 (6.6)		240 (6.7)	
**CPR, n (%)**								
	2018	674 (28.8)	338 (27.4)		851 (29.7)		1863 (28.9)	
	2019	872 (36.4)	532 (40.5)		1215 (39)		2619 (38.3)	
	2020	1095 (37.5)	605 (40.2)		1502 (41.2)		3202 (39.7)	
	2021	1147 (40.8)	677 (46.1)		1557 (45.7)		3381 (44)	
	2022	1715 (46.8)	965 (49)		2411 (49.3)		5091 (48.4)	
	2023	601 (46.1)	291 (47.6)		752 (45.4)		1644 (46)	

^a^OHCA: out-of-hospital cardiac arrests.

^b^STA: survival to admission.

^c^CPR: cardiopulmonary resuscitation.

^d^HK: Hong Kong.

^e^KW: Kowloon.

^f^HKI: Hong Kong Island.

^g^NT: New Territories and Outlying Islands.

### Relationship Between STA and Patients’ Characteristics

Table 3 presents the results of the multivariate logistic regression analysis of the associations between the STA rate and various factors during the prepandemic and pandemic periods. The key findings included higher odds of OHCA among men during the pandemic period and higher odds of certain initial electrocardiogram rhythms during the same period. There were also higher odds of nontrauma causes of OHCA during both periods. There were other factors, such as the response time, whether the OHCA was witnessed, the location of the OHCA, and the region exhibited varying aORs between the 2 periods. Notably, we found that bystander CPR administration (aOR 1.48, 95% CI 1.17-1.86) and PAD use (aOR 1.16, 95% CI 1.05-1.28) were significantly associated with a high STA rate during the pandemic period.

**Table 3:** The associations of STA^a^ and patient’s characteristics.

Characteristics		Prepandemic period	Pandemic period
		Adjusted OR^b^ (95% CI)	Adjusted OR (95% CI)
**Sex**			
	Female	—^c^	—
	Male	0.95 (0.87-1.05)	1.12^*^ (1.02-1.24)
Age (years)		0.99^***^ (0.99-0.99)	0.99^***^ (0.99-0.99)
**Cause of arrest**			
	Trauma	—	—
	Nontrauma	4.35^***^ (3.10-6.10)	4.48^***^ (3.05-6.56)
**Initial electrocardiogram rhythm**			
	Asystole	—	—
	VF/VT^d^	2.92^***^ (2.49-3.43)	3.49^***^ (3.03-4.01)
	PEA^e^	2.31^*^ (2.05-2.61)	2.91^***^ (2.60-3.25)
	Response time	0.99 (0.98-1.00)	0.97^***^ (0.96-0.98)
**Witnessed arrest**			
	Yes	—	—
	No	0.48^***^ (0.43-0.53)	0.49^***^ (0.45-0.54)
**Location of OHCA^f^**			
	Street	—	—
	Home	0.47^***^ (0.37-0.58)	0.59^***^ (0.48-0.72)
	En route to a hospital	0.78 (0.58-1.05)	1.56^***^ (1.19-2.05)
	Home for aged	0.45^***^ (0.36-0.58)	0.53^***^ (0.42-0.67)
	Public area excluding street	0.75^*^ (0.59-0.95)	0.97 (0.78-1.20)
**Region**			
	NT^g^	—	—
	HKI^h^	0.78 (0.68-0.88)	0.75^***^ (0.66-0.85)
	KW^i^	0.66^***^ (0.60-0.74)	0.86^**^ (0.78-0.95)
**Bystander PAD^j^**			
	No	—	—
	Yes	1.14 (0.86-1.51)	1.48^**^ (1.17-1.86)
**Bystander CPR^k^**			
	No	—	—
	Yes	1.04 (0.93-1.16)	1.16^**^ (1.05-1.28)
Pseudo *R*^2^		0.102	0.125
Akaike information criterion		11,597.9	14,440.9
Bayesian information criterion		11,725.9	14,581.6

^a^STA: survival to admission.

^b^OR: odds ratio.

^c^Not applicable.

^d^VF/VT: ventricular fibrillation or ventricular tachycardia.

^e^PEA: pulseless electrical activity.

^f^OHCA: out-of-hospital cardiac arrests.

^g^NT: New Territories and Outlying Islands.

^h^HKI: Hong Kong Island.

^i^KW: Kowloon.

^j^PAD: public-access defibrillation.

^k^CPR: cardiopulmonary resuscitation.

*<.05, **<.01, ***<.001.

### Correlation Between OHCA and COVID-19 Cases During the Pandemic

**Figure 3:**
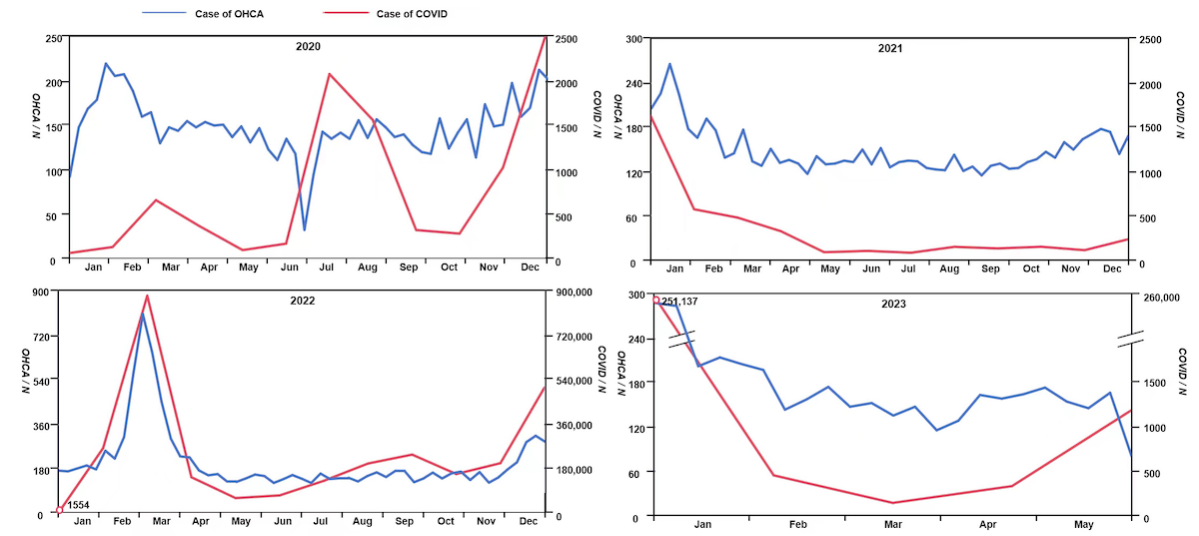
Correlation of prevalence between OHCA and COVID-19 during pandemic (January 2020 to May 2023). OHCA: out-of-hospital cardiac arrests.

Figure 3 graphically presents the correlation between OHCA incidents and the progression of COVID-19 during the pandemic period in HK. Overall, there was a consistent relationship between these 2 variables, with a higher SARS-CoV-2 infection rate leading to an increased prevalence of OHCA. This correlation was particularly evident in 2022, which had the highest prevalence of COVID-19 and corresponded with the highest prevalence of OHCA. Table 4 provides a detailed overview of the OHCA and COVID-19 statistics from February 7, 2022, to April 24, 2022. This period marked the peak of COVID-19 cases, which coincided with a sharp increase in the number of OHCA incidents. During these 11 weeks, the number of OHCA cases increased dramatically, reaching a peak of approximately 114 cases per day in the fourth week. Concurrently, the number of COVID-19 cases increased, reaching a daily average of 67,583 cases during the same week. However, the highest daily death toll from COVID-19 was recorded in the fifth week. In contrast, the STA rate in cases of OHCA demonstrated a slightly different trend, with marginally higher numbers recorded during the most severe 5 weeks (weeks 3-7) of the COVID-19 outbreak.

**Table 4:** OHCA^a^ and COVID-19 cases in the peak (weeks) of the pandemic period in HK^b^.

Number	Weeks	OHCA cases, median (IQR)	STA^c^ of OHCA, median (IQR)	COVID-19 cases, median (IQR)	Daily death of COVID-19, median (IQR)
1	February 7, 2022, to February 13, 2022	30 (26-38)	1 (0-1)	1161 (625-1347)	0 (0-2)
2	February 14, 2022, to February 20, 2022	47 (38-50)	1 (1-2)	4285 (2071-6067)	11 (3-17)
3	February 21, 2022, to February 27, 2022	86 (64-93)	2 (1-4)	8798 (7533-50,976)	49 (36-80)
4	February 28, 2022, to March 6, 2022	114 (104-126)	2 (1-3)	67,583 (55,658-76,341)	203 (183-238)
5	March 7, 2022, to March 13, 2022	94 (90-98)	3 (2-4)	30,493 (27,647-32,430)	290 (285-296)
6	March 14, 2022, to March 20, 2022	63 (54-80)	2 (0-3)	21,650 (16,597-27,765)	284 (254-294)
7	March 21, 2022, to March 27, 2022	42 (38-45)	3 (1-3)	12,240 (8841-14,068)	206 (156-228)
8	March 28, 2022, to April 3, 2022	32 (31-36)	2 (1-4)	6646 (4475-7596)	120 (116-156)
9	April 4, 2022, to April 10, 2022	30 (27-36)	2 (0-3)	2644 (2492-3138)	87 (65-97)
10	April 11, 2022, to April 17, 2022	22 (19-30)	1 (1-3)	1043 (794-1407)	57 (41-62)
11	April 18, 2022, to April 24, 2022	21 (17-26)	1 (1-2)	600 (523-628)	15 (10-20)

^a^OHCA: out-of-hospital cardiac arrests.

^b^HK: Hong Kong.

^c^STA: survival to admission.

## Discussion

### Primary Findings

Data from the past 7 years show that, during the COVID-19 pandemic, the number of OHCA cases in HK increased significantly, almost quadrupling at the peak of the pandemic. Concurrently, the survival rate in going to a hospital for OHCA cases decreased considerably, leading to a marked increase in OHCA-related deaths. This trend slowly decreased to normal levels by the end of this study’s period. Although fatalities directly attributed to COVID-19 may account for some of these findings, it is crucial to acknowledge the potential influence of various indirect effects. For instance, the widespread implications of lockdowns led to significant escalations in stress and anxiety levels, which may have increased the likelihood of OHCA [9]. Another critical factor is the strain on the health care system owing to the pandemic, such as the EMS being overwhelmed. Such disruptions adversely affect all components of the chain of survival [10]. However, without data on the SARS-CoV-2 infection rate in the OHCA population, it is challenging to estimate the direct impact of COVID-19 on the STA rates of patients with OHCA.

### Comparisons With Previous Studies

During the pandemic, a significant shift in OHCA location was observed. Compared to before the pandemic, during the pandemic there was a decrease in the number of OHCAs occurring in public places, likely due to the implementation of lockdowns and social distancing measures, which resulted in fewer people being out in public. This finding aligns with those of most previous studies [11-13], but a different trend has been reported in some Asian populations. For instance, Riyapan et al [14] reported no significant change in the occurrence of OHCA in public places in Thailand before and during the pandemic. Watanabe et al [15] also reported no decrease in the occurrence of OHCA in public places. However, the decrease observed in the number of OHCAs in public places does not necessarily indicate a reduction in the overall number of OHCAs. Instead, this may suggest a shift in the location of these incidents, with a potential increase in the number of OHCAs occurring at home.

There was a significant decrease in the number of OHCAs reported in nursing homes during the pandemic. This trend is surprising given the vulnerability of the older population to OHCA. The findings for this outcome in previous studies have been mixed. For example, a study in Portugal reported no difference between the number of OHCAs in nursing homes before and during the pandemic [13]. Another study in the United States found that the percentage of patients experiencing an OHCA in a nursing home was higher in 2020 than in 2019 [16]. A study in HK revealed that 96% of COVID-19–related deaths were among individuals aged 60 years and older, with 53% being nursing home residents [17]. Our findings suggest that, although the number of OHCAs in nursing homes decreased during the pandemic, this does not necessarily mean fewer older individuals experienced an OHCA. Many older individuals may have died from COVID-19, and their deaths were not classified as OHCA-related. This may result in an underestimation of the devastating impact of OHCA on the older population, particularly for those in nursing homes, during this public health emergency. The government should update and standardize mortality calculations during future public health emergencies [18].

Another reason for the decrease in the number of OHCAs in nursing homes during the pandemic may be the effectiveness of nonpharmaceutical interventions in reducing the number of COVID-19 cases and fatalities within long-term care facilities and nursing homes. Previous studies have shown an association between a decrease in the number of COVID-19 cases and the rigor of various containment measures, notably school and workplace closures, and public information campaigns globally [19,20]. In HK, border restrictions, quarantine and isolation, social distancing, and behavioral changes in the population were significantly linked to the control of the COVID-19 pandemic [21]. This may underscore the crucial role that nonpharmaceutical interventions can play in safeguarding vulnerable populations during pandemics.

Our study revealed an increase in the bystander CPR rate during both the prepandemic and pandemic periods, albeit at a slower rate during the pandemic. During the prepandemic period, the bystander CPR rate increased by approximately 10% from 2018 to 2019. However, this trend plateaued with the onset of the COVID-19 pandemic, resulting in a negligible difference in the CPR rate between 2019 and 2020. A Korean study reported a similar finding [22], but studies in some Western countries have reported notable decreases in CPR rates. For example, in France, the CPR rate dramatically fell from 63.9% to 47.8% between March and April 2020 [12]. A study in Spain also found a decrease in CPR rates from 51.1% to 42.6% between 2017/2018 and 2020 [23]. These decreases in CPR rates highlight the profound effects of the pandemic on critical medical procedures across different nations. Nevertheless, we found that, as the pandemic progressed, the CPR rate showed another increase, stabilizing between 44% and 48%. Data on bystander CPR rates during the pandemic are mixed. A systematic review of studies conducted in 2020 suggested a decline in the incidence of bystander CPR at the onset of the pandemic, with 1 study noting that this decline was observed only in patients diagnosed with COVID-19 [24]. Another review, including studies published in 2020 and 2021, indicated a change in the community response to OHCA, with fewer occurrences of bystander CPR [25]. However, a recent review of studies published in 2022 found no significant difference in bystander CPR rates between the COVID-19 pandemic and prepandemic periods [4]. It is important to note that the pandemic also affected other aspects of the CPR response and outcomes, such as EMS availability. A review of the relationship between OHCA and EMS indicated that difficulties encountered by the first responder system during the COVID-19 pandemic, such as dispatcher overload, increased response times, and adherence to personal protective equipment requirements, were superimposed on shortages in the supply of personal protection equipment [26]. Our findings highlight the complex impact of the pandemic on OHCA outcomes.

Although the frequency of witnesses of OHCA and bystander CPR significantly increased, we observed a drastic reduction in the rate of STA en route to the A&E department during the pandemic, which is consistent with the findings of previous studies [4,24,25]. During the prepandemic period, the overall STA rate in 2018 was approximately 16.9%, and this increased slightly to 18.5% in 2019. However, during the first year of the pandemic, the STA rate decreased by more than 25% and further decreased to approximately 5.6% at the peak of the pandemic in 2022. By the end of this study’s period, the STA rate had slightly increased to 6.7% in the first 5 months of 2023 but remained approximately 65% lower than the rate observed in the final year of the prepandemic period (2019). The STA rate was lower in HK than in other countries or regions during the pandemic [12,27,28]. Several factors may have accounted for this discrepancy. An overburdened health care system can lead to delayed recognition of and intervention in unstable patients. For instance, following the 2022 Chinese New Year holiday, public hospitals in HK were dealing with more than 1800 COVID-19 cases requiring EMS per day. Emergency medical systems, especially emergency departments, which serve as the frontline of the health care system, experienced severe overload and challenging working conditions. This was a global issue [29]. Moreover, there were fewer cardiac arrests in public places during the pandemic, which may have influenced the survival rate of patients with OHCA. Additionally, the lack of sufficient CPR training in HK [30,31] and the fear of COVID-19 may have affected the willingness of witnesses to perform CPR. Given that CPR is considered an aerosol-generating procedure with a significant risk of viral transmission, witnesses may have hesitated to perform it, potentially affecting the STA rate [32].

A strong correlation was observed between outbreaks of COVID-19 and surges in OHCA cases in HK over the past 4 years. In particular, the fifth wave of the pandemic, which coincided with the Chinese New Year holiday, resulted in a significant surge in OHCA cases. The older population, the most vulnerable group to both COVID-19 and OHCA, bore the brunt of the pandemic. The average age of our sample was over 75 years (SD 17.1 years), highlighting the insufficient protection provided by the government for this demographic during the pandemic. A previous review demonstrated a gradual age-associated increase in the COVID-19 mortality rate [33]. For example, 1 study showed a 10.5% fatality rate for older patients compared to 0.43% for younger patients [34]. As of April 2022, less than one-third of HK residents aged older than 80 years had been fully vaccinated. The vaccination rate was even lower among residents of care homes when the Omicron variant arrived in HK, at less than 20%. This is not surprising, given that vaccine hesitancy to both the first vaccine dose and the booster was widespread [35]. Another study underscored the urgency of vaccination for older people, revealing that approximately one-quarter of countries reported lower vaccination coverage among older adults compared to the overall population [36]. Additionally, as reported in a previous study [37], lockdown measures and no-visit policies resulted in older adults being unable to meet their families for extended periods, leading to heightened levels of anxiety and depression among this population, thereby increasing the risk of OHCA. It is crucial to develop comprehensive policies and strategies to mitigate the direct and indirect effects of public health emergencies on health outcomes in the older population.

In 2019, the HK Legislative Council suggested promoting bystander intervention to improve the survival rates for patients with OHCA. The suggestions included adopting technologies to reduce rescue time, providing education and training to enhance the skills of the public, and urging the government to develop laws and regulations to protect bystanders legally and limit their liability [38]. Thus, the outcomes of our regression analysis revealed that, during the pandemic, bystander CPR and PAD use significantly improved the STA rate for individuals with OHCA compared to the prepandemic period, during which the associations between STA rate and bystander interventions were insignificant. Furthermore, our regression analysis revealed a significant relationship between the response time and STA rate. To handle emergencies during the pandemic, the HKFSD adopted a target-oriented approach to devise various emergency response strategies and measures. In the meantime, the Director’s Command Post also established an artificial intelligence–powered social media service platform capable of providing automated inquiries and instant replies 24 h/d, 7 d/wk [39]. The importance of reform and collaboration in multiple social sectors is paramount to improving the STA rate of patients with OHCA during public health emergencies.

### Limitations

Our study is among the first to delineate the relationship between COVID-19 and the prevalence of OHCA at the population level during both the prepandemic and pandemic periods. However, this study has certain limitations that need to be acknowledged. First, although we observed a decrease in the STA rate during the pandemic, we lacked information about patient survival rates upon discharge and their short- and long-term health outcomes, because patient data in A&E departments and hospital settings are managed by the Hospital Authority rather than the HKFSD. Matching OHCA patient data with medical records would provide a more comprehensive understanding of the impact of COVID-19 on OHCA in HK. Second, the HKFSD did not collect information about whether patients with OHCA had COVID-19 during the provision of services. Consequently, we lack knowledge about the prevalence of COVID-19 among patients with OHCA, which may have led to an inaccurate estimation of the impact of COVID-19 on the survival rate of patients with OHCA in HK.

### Conclusions

In our study of the prevalence of OHCA from 2017 to 2023, we observed a significant increase in OHCA incidence and a considerable decrease in survival rates of patients with OHCA during the COVID-19 pandemic compared to the period before the pandemic. Moreover, we found that the spikes in the number of OHCA cases closely matched the increase in the number of confirmed COVID-19 cases in HK. Components of both the social sector, such as the Home Office, and the health care sector, such as EMS systems, should prepare for potential increases in the number of OHCA cases during future public health crises. It is necessary to reform these sectors to make them more resilient, efficient, and effective at meeting the needs of individuals with OHCA. Developing an effective multidisciplinary mechanism for leveraging resources, sharing knowledge and expertise, and coordinating efforts are key factors for improving the survival rates of patients with OHCA during future pandemics.

## Appendix

Multimedia Appendix 1 Out-of-hospital cardiac arrest incidence, survival to admission, and cardiopulmonary resuscitation ratio in different demographic and geographic groups.

## Abbreviations

A&E: accident and emergency
aOR: adjusted odds ratio
CHP: Centre for Health Protection
CPR: cardiopulmonary resuscitation
EMS: emergency medical service
HK: Hong Kong
HKFSD: Hong Kong Fire Service Department
OHCA: out-of-hospital cardiac arrest
PAD: public-access defibrillator
STA: survival to admission

## Data Availability

The data sets generated or analyzed during this study are not publicly available due to privacy concerns. However, they may be obtained from the corresponding author upon reasonable request.
