# Supplementary material for: Out-of-Hospital Cardiac Arrest Before and During the COVID-19 Pandemic in Hong Kong: Registry-Based Study From 2017 to 2023
Source: JMIR Public Health Surveill. 2024 May 21;10:e56054. doi: 10.2196/56054 (PMC11150895; doi:10.2196/56054)
Supplement: Multimedia Appendix 1 [file publichealth_v10i1e56054_app1.docx]

**Multimedia Appendix 1.** Table OHCA incidence, STA, and CPR ratio in different demographic and geographic groups. CPR: cardiopulmonary resuscitation; OHCA: out-of-hospital cardiac arrest; STA: survival to admission.

| **OHCA, N (Incidence rate, %)** | **2018** | **2019** | **2020** | **2021** | **2022** | **2023** |
| --- | --- | --- | --- | --- | --- | --- |
| **Sex** |  |  |  |  |  |  |
| Male | 3635(56.5) | 3912(57.3) | 4460(55.3) | 4320(56.2) | 5943(56.1) | 1998(55.6) |
| Female | 2803(43.5) | 2919(42.7) | 3608(44.7) | 3371(43.8) | 4643(43.9) | 1593(44.4) |
| **Age groups** |  |  |  |  |  |  |
| ≤40 | 250(3.9) | 265(3.9) | 287(3.6) | 589(7.7) | 418(4) | 101(2.9) |
| 41-64 | 1284(20.2) | 1341(19.9) | 1428(17.8) | 1342(17.4) | 1583(15.2) | 637(18.4) |
| 65-74 | 919(14.4) | 1022(15.2) | 1290(16.1) | 1199(15.6) | 1589(15.2) | 554(16) |
| 75-84 | 1490(23.4) | 1461(21.7) | 1693(21.2) | 1462(19) | 2104(20.2) | 680(19.7) |
| ≥85 | 2418(38) | 2653(39.4) | 3305(41.3) | 3104(40.3) | 4740(45.4) | 1484(42.9) |
| **Regions**(Incidence rate, ‰) |  |  |  |  |  |  |
| KW | 2338 (1.03) | 2398(1.04) | 2922(1.27) | 2818(1.26) | 3695(1.68) | - |
| HKI | 1232(0.98) | 1314(1.05) | 1504(1.23) | 1468(1.23) | 1978(1.71) | - |
| N.T. | 2868(0.73) | 3119(0.79) | 3642(0.92) | 3410(0.86) | 4945(1.24) | - |
|  |  |  |  |  |  |  |
| **STA, N (%)** | **2018** | **2019** | **2020** | **2021** | **2022** | **2023** |
| **Sex** |  |  |  |  |  |  |
| Male | 681(18.7) | 761(19.5) | 692(15.5) | 365(8.5) | 396(6.8) | 158(7.9) |
| Female | 409(14.6) | 500(17.1) | 407(11.3) | 176(5.2) | 185(4) | 82(5.1) |
| **Age groups** |  |  |  |  |  |  |
| ≤40 | 55(22) | 50(18.9) | 40(13.9) | 55(9.4) | 28(6.8) | 6(5.9) |
| 41-64 | 288(22.4) | 335(25) | 290(20.3) | 147(11) | 141(9.1) | 70(11) |
| 65-74 | 203(22.1) | 226(22.1) | 230(17.8) | 100(8.4) | 131(8.4) | 45(8.1) |
| 75-84 | 247(16.6) | 264(18.1) | 251(14.8) | 90(6.2) | 122(5.9) | 48(7.1) |
| ≥85 | 296(12.2) | 385(14.5) | 286(8.7) | 149(4.8) | 145(3.1) | 60(4) |
| **Regions** |  |  |  |  |  |  |
| KW | 342(14.6) | 369(15.4) | 348(11.9) | 212(7.6) | 215(5.9) | 99(7.5) |
| HKI | 196(15.9) | 225(17.1) | 181(12.0) | 84(5.7) | 92(4.7) | 31(5.0) |
| N.T. | 552(19.2) | 667(21.4) | 570(15.7) | 245(7.2) | 277(5.7) | 110(6.6) |
|  |  |  |  |  |  |  |
| **CPR, N (%)** | **2018** | **2019** | **2020** | **2021** | **2022** | **2023** |
| **Sex** |  |  |  |  |  |  |
| Male | 1043(28.7) | 1452(37.1) | 1734(38.9) | 1859(43.1) | 2761(46.9) | 904(45.6) |
| Female | 820(29.3) | 1167(40) | 1468(40.7) | 1520(45.1) | 2319(50.3) | 735(46.6) |
| **Age groups** |  |  |  |  |  |  |
| ≤40 | 70(28) | 99(37.4) | 111(38.7) | 232(39.5) | 169(40.9) | 40(40) |
| 41-64 | 362(28.2) | 508(37.9) | 583(40.8) | 589(43.9) | 733(46.8) | 283(44.9) |
| 65-74 | 239(26) | 361(35.3) | 472(36.6) | 540(45.2) | 746(47.2) | 247(45.1) |
| 75-84 | 403(27) | 560(38.3) | 669(39.5) | 642(43.9) | 987(47.4) | 304(45.2) |
| ≥85 | 768(31.8) | 1070(40.3) | 1350(40.8) | 1378(44.4) | 2394(50.9) | 717(48.6) |
| **Regions** |  |  |  |  |  |  |
| KW | 674(28.8) | 872(36.4) | 1095(37.5) | 1147(40.8) | 1715(46.8) | 601(46.1) |
| HKI | 338(27.4) | 532(40.5) | 605(40.2) | 677(46.1) | 965(49.0) | 291(47.6) |
| N.T. | 851(29.7) | 1215(39.0) | 1502(41.2) | 1557(45.7) | 2411(49.3) | 752(45.4) |
